# Cancer prevention in Germany: awareness, beliefs, and information-seeking behaviors in a population-based survey

**DOI:** 10.3389/fpubh.2026.1868556

**Published:** 2026-06-30

**Authors:** Mona Illmann, Karen Steindorf, Alexander Haussmann, Florian Herbolsheimer

**Affiliations:** 1 Division of Physical Activity, Cancer Prevention and Survivorship, German Cancer Research Center (DKFZ), Heidelberg, Germany; 2 Faculty of Medicine, Heidelberg University, Heidelberg, Germany; 3 Faculty of Medicine, Medical Center, Section of Health Care Research and Rehabilitation Research, Institute of Medical Biometry and Statistics, University of Freiburg, Freiburg, Germany

**Keywords:** cancer awareness, cancer myths, cancer prevention, information overload, information seeking

## Abstract

**Introduction:**

At least 40% of cancer cases could be prevented by lifestyle changes. However, despite clear recommendations, public awareness of modifiable cancer risk factors remains limited and misconceptions about cancer causes further impede prevention efforts. Understanding knowledge of preventable factors, myths, and information sources is essential for effective cancer prevention.

**Methods:**

To address this, a total of 1,232 residents of Stuttgart, Germany (53.2% female; *M_age_* = 41.1; *SD* = 14.3) participated in the CLARO study (January–May 2025). A cross-sectional online survey assessed participants’ knowledge of cancer risk factors and myths, cancer information overload, and sources of prevention information.

**Results:**

Smoking (98.5%) and sunburn (95.1%) were widely recognized as cancer risk factors, whereas high salt intake (28.1%) and prolonged sitting (29.2%) were less acknowledged. Most cancer myths went unrecognized, except physical trauma (76.9%). Higher levels of cancer risk factor knowledge were linked to higher education (middle vs. high: *β* = −0.069, *p* = 0.022; low vs. high: *β* = −0.053, *p* = 0.048), prior cancer prevention information retrieval (*β* = 0.186, *p* < 0.001), and less cancer information overload (*β* = −0.235, *p* < 0.001).

**Discussion:**

Persistent knowledge gaps and misconceptions highlight the need to promote access to trusted, expert-reviewed information sources.

## 1 Introduction

Cancer remains one of the leading causes of morbidity and mortality worldwide (1). In Germany, an estimated 504,000 new cancer cases were diagnosed in 2022 (2) and cancer was the second leading cause of death in 2024 (3). At the same time, estimates for Germany suggest that approximately 37.4% of cancer cases are attributable to preventable risk factors (4). Comparable estimates have also been reported globally (5). Therefore, the 4th edition of the European Code Against Cancer (ECAC4) outlines 12 evidence-based lifestyle recommendations designed to advise individuals to reduce their cancer risk. These include, among others, avoiding tobacco use, maintaining a healthy body weight, engaging in regular physical activity, adhering to a balanced diet, participating in recommended vaccination programs, and limiting alcohol consumption (6). Accurate public awareness of these behaviors is crucial for making informed decisions about lifestyle behavior changes that can reduce one’s cancer risk.

International studies examining public awareness of cancer risk behaviors report varying levels of knowledge across countries. The most consistent finding is the high public awareness for smoking (59.2–100%) (7–9) as a risk factor. In contrast, the recognition of other modifiable lifestyle-related risks remains substantially lower. These include the awareness of the cancer risk reduction potential of physical activity (28.0–60.5%) (10–12), as well as awareness of the cancer risk increase potential associated with overweight and obesity (29.0–66.9%) (7, 8, 12), low fruits and vegetables consumption (23.0–86.1%) (8, 9, 11), the intake of red and/or processed meats (31.0–80.7%) (8, 11, 12), alcohol consumption (35.0–86.8%) (8, 10, 12), and human papillomavirus (HPV) infection (36.0–57%) (9, 13, 14).

Beyond well-established risk factors, public perceptions of cancer risk frequently include widespread misconceptions. A substantial proportion of individuals believe that factors not supported by scientific evidence, e.g., exposure to electromagnetic frequencies or consumption of food containing artificial additives, are linked to cancer development (9, 13). Such misconceptions may shift focus away from established, modifiable risk factors and thereby undermine effective prevention efforts (15). Another factor contributing to limited awareness of established cancer risk factors is cancer information overload, a state in which individuals feel overwhelmed by the amount of cancer-related material in the information environment (16), often amplified by the internet and newspapers (17, 18). Correspondingly, between 63.1 and 74.7% of participants in previous studies agreed with the statement “There are so many recommendations about preventing cancer, it’s hard to know which ones to follow” (19–22). This sense of overload has been linked to reduced engagement in cancer prevention behaviors, including lower colonoscopy uptake (16), reduced attendance at annual medical checkups (23) and less favorable dietary choices (24). Moreover, cancer information overload is associated with increased cancer information avoidance (23, 25, 26).

In this context, it is crucial to understand not only public beliefs about cancer risk, but also the sources from which individuals obtain their information. A comprehensive understanding of public knowledge, beliefs, and perceptions regarding cancer risk factors is critical for developing effective, evidence-based health promotion strategies aimed at addressing knowledge gaps and facilitating behavior change. Equally important is an examination of where individuals obtain information on cancer prevention, as well as the factors that influence whether they trust and act upon such information, for example the perceived credibility of the source. Previous research has shown that the use of reliable sources, such as websites of public institutions and healthcare providers, are associated with a wide range of favorable health behaviors (27–29). These findings underscore the importance of identifying the types of information sources participants use, as they represent critical determinants of both knowledge acquisition and health-related decision-making.

However, to date, no comprehensive, population-based assessment has been conducted in Germany to evaluate public knowledge of cancer risk factors, information overload, or patterns of information-seeking behavior in this context. To address this gap, the present study aimed to: (1) assess knowledge of evidence-based and mythical cancer risk factors, cancer information overload, and cancer prevention-related information-seeking behaviors; and (2) examine associations among these variables.

## 2 Materials and methods

### 2.1 Participant recruitment and data collection

A list of 6,000 randomly selected residents from Stuttgart, a large city in Germany, was obtained from the residents’ registration office. To account for the anticipated lower response rate among socially and economically disadvantaged groups, individuals from districts with lower average socioeconomic status were over-sampled (see Supplementary material). Between January 30 and May 31, 2025, persons were invited by letter to participate in the cross-sectional online survey.

Participants were informed about the aims of the study and its voluntary nature in the invitation letter, and provided written informed consent as part of the online survey, which was implemented using LimeSurvey (Limesurvey GmbH, Hamburg, Germany). Upon request, participation via a paper-and-pencil version of the survey was also possible. Participants had to be between 18 to 70 years and be able to speak and understand German. Participants were offered an incentive of 10€ upon completing the survey.

To ensure data quality, two seriousness checks were embedded in the online survey. The first was an attention check included within a multiple-choice question: “If you are reading this carefully, please select option 2”. The second was a self-assessment item at the end of the questionnaire: “In your honest opinion, should we use your data? This question refers to whether you answered all questions seriously”. Participants were explicitly informed that their response to this item would not affect their compensation.

This study was conducted as part of the CLARO project. A protocol for the study was registered and published online at ClinicalTrials.gov (identifier: NCT06614673) before the data collection started. Ethics approval for this study was granted by the Ethics Committee of the Medical Faculty of Heidelberg University (S-512/2022).

### 2.2 Measures

Participants completed an online survey comprising various questionnaires designed to collect a broad range of information, including the following measures.

#### 2.2.1 Socio-demographics and cancer prevention behaviors

Participants were asked to provide sociodemographic information, including age, gender, marital status, highest attained level of education and vocational training, current occupational status, and household income. Educational attainment was categorized according to the International Standard Classification of Education (ISCED-11) (30) and subsequently divided into lower (primary and lower secondary education; levels 1–2), middle (upper secondary education; level 3) and higher (post-secondary non-tertiary education and above; levels 4–8) education levels. In addition, participants were asked whether they or someone with whom they had direct contact with (e.g., a close family member, partner, friend, colleague, or patients encountered at work) had ever received a cancer diagnosis.

Engagement in preventive behaviors was assessed using single-item measures for each behavior, based on previous studies (13, 31). Smoking status was assessed with response options ranging from never to daily smoking, including former and other tobacco use. Fruit and vegetable consumption was assessed by the number of days in the past week participants consumed at least five portions; respondents consuming at least five portions per day were classified as meeting current guidelines (32). Physical activity was measured by the number of days participants engaged in ≥ 30 min of at least moderate-intensity activity; participants active on ≥ 5 days per week - equivalent to at least 150 min - were classified as meeting guidelines (33). Body mass index (BMI) was calculated from self-reported height and weight and categorized as underweight (< 18.5), normal weight (18.5–24.9), overweight (25–29.9), or obese (≥ 30).

#### 2.2.2 Cancer prevention knowledge

Awareness of cancer risk factors was assessed using the validated Cancer Awareness Measure (CAM) (34, 35). Twelve evidence-based risk factors not covered by the CAM, but included in the ECAC4 (6), were additionally incorporated. To assess misconceptions, the Mythical Causes Scale (CAM-MYCS) was used (36), which includes commonly believed but scientifically unsubstantiated causes of cancer. In total, participants were presented with 38 potential cancer risk factors - 26 evidence-based and 12 mythical - in randomized order and asked whether they believed each factor could increase a person’s risk of cancer. Response options were “yes,” “no,” or “don’t know/not sure.” Responses were scored as correct or incorrect based on current scientific consensus, with “don’t know/not sure” categorized as incorrect. Knowledge scores were calculated by summing the number of correct responses.

#### 2.2.3 Cancer information overload

In line with previous studies (21, 37), cancer information overload was assessed using a single item from the National Cancer Institute’s Health Information National Trends Survey (38): “There are so many recommendations about preventing cancer, it’s hard to know which ones to follow.”. The item was rated on a 4-point Likert scale ranging from 1 (“strongly agree”) to 4 (“strongly disagree”) and subsequently dichotomized into “agree” (strongly agree/agree) and “disagree” (strongly disagree/disagree).

#### 2.2.4 Information retrieval on cancer prevention

Information retrieval on cancer prevention - defined as the extent to which individuals actively seek or access information related to cancer risk reduction - was assessed using three additional items from the CAM toolkit (35). Participants were asked whether they had ever sought information on cancer prevention. If they answered “yes,” they were then asked about (1) the sources of information they had used (8 response options) and (2) the features of those sources that influenced their decision to use them (11 response options). If they answered “no,” they were asked about (1) the sources they would consider using (8 response options) and (2) the features that would influence their choice (11 response options).

### 2.3 Statistical analysis

Normality of continuous variables was assessed using Shapiro–Wilk tests and visual inspection of the distribution. Group differences were then examined using paired Wilcoxon signed-rank tests or Wilcoxon rank-sum tests when normality assumptions were violated. Specifically, a paired Wilcoxon test compared knowledge of actual cancer risk factors versus cancer myths, as the distribution of the cancer myths knowledge score deviated from normality. A Wilcoxon rank-sum test was used to compare age between participants who had previously sought cancer prevention information and those who had not, since age was not normally distributed. Bivariate associations were tested using Spearman correlations when variables deviated from normality.

Predictors of knowledge outcomes were examined using generalized linear models. Multiple linear regression was applied to test associations between knowledge of actual cancer risk factors and predictors including age, gender, education, prior cancer prevention information retrieval, cancer information overload, and personal cancer experience, with robust standard errors applied to account for heteroscedasticity. To assess predictors of cancer myths knowledge scores, a negative binomial regression model was conducted due to overdispersion, including the same set of predictors, with model fit compared against a Poisson model. Assumptions of linearity, normality of residuals, and homoscedasticity were checked prior to regression analyses. Normality of residuals was inspected using Q-Q plots and Shapiro–Wilk tests.

All statistical analyses were conducted using R version 4.4.2 (39) and statistical significance was evaluated at the *α* = 0.05 level.

## 3 Results

### 3.1 Sample characteristics

Of the 6,000 individuals invited to participate, 1,418 took part in the survey (including 8 via paper-and-pencil format). After excluding 88 participants who discontinued the survey prematurely and 98 who failed seriousness checks, the final sample consisted of *N* = 1,232 participants (see Supplementary Figure S1 for a participant flowchart of the recruitment process). The mean age was 41.1 years (*SD* = 14.3), with a slight majority identifying as female (53.2%) and most reporting current employment (67.5%). Descriptive statistics on participants’ sociodemographic characteristics, health-related behaviors, and cancer information overload are presented in Table 1.

**Table 1:** Sociodemographic characteristics, health-related behaviors and cancer information overload of the sample.

Sociodemographic characteristics	*N* (%)	Health-related behaviors and cancer information overload	*N* (%)
Gender		Smoking cigarettes*	
Female	655 (53.2)	Never	691 (56.1)
Male	573 (46.5)	Not anymore	318 (25.8)
Diverse	4 (0.3)	Not every day	89 (7.2)
Age (Mean ± SD)	41.1 ± 14.3	Every day	98 (8.0)
18–24 years	160 (13.0)	Other tobacco product	28 (2.3)
25–34 years	329 (26.7)	Guideline adherence physical activity^**a,c**^	
35–49 years	355 (28.8)	Yes	415 (33.8)
50–64 years	304 (24.7)	No	801(65.0)
65–70 years	84 (6.8)		
Employment status^**a**^		Guideline adherence to fruits and vegetables consumption^**a,c**^	
Employed	832 (67.5)	Yes	303 (24.6)
Housework	23 (1.9)	No	893 (72.5)
Retired	91 (7.4)	BMI (Mean ± SD)	24.51 ± 4.42
On sick leave	15 (1.2)	Underweight <18.5	39 (3.2)
Unemployed	46 (3.7)	Normal 18.5–24.9	728 (59.1)
In education	180 (14.6)	Overweight 25–29.9	320 (26.0)
Education^**a,**b^		Obese ≥ 30	131 (10.6)
Lower	45 (3.7)	Personal experience with cancer^**a**^	
Middle	352 (28.6)	Own experience	51 (4.1)
Higher	834 (67.7)	Family member	625 (50.7)
Monthly household income^**a**^		Partner	40 (3.2)
<1,000€	83 (6.7)	Friend	364 (29.5)
1,000–2000€	110 (8.9)	Co-worker	399 (32.4)
2000–3,000€	179 (14.5)	Work	122 (9.9)
3,000–4,000€	192 (15.6)	None of the above	251 (20.4)
4,000–5,000€	134 (10.9)	CIO: There are so many recommendations about preventing cancer, it’s hard to know which ones to follow. ^**a,d**^	
>5,000€	368 (29.9)	Disagree	597 (48.5)
Family status		Agree	550 (44.6)
Not married	607 (49.3)		
Married	527 (42.8)		
Divorced/separated	85 (6.9)		
Widowed	13 (1.1)		

*N* = 1,232.

^**a**^The total number is different from 1,232 because of missing data. Percentages are based on the total *N*.

^b^Education: Educational attainment was categorized according to the International Standard Classification of Education (ISCED-11): Lower = ISCED-11 levels 1–2, Middle = ISCED-11 level 3, Higher = ISCED-11 levels 4–8.

^**c**^ Health related behaviors.

Guideline adherence to fruits and vegetables consumption: ≥5 servings of fruits and vegetables daily over the past week Guideline adherence physical activity: ≥5 days with ≥30 min of physical activity in the past week.

^**d**^ Responses on the 4-point Likert scale were dichotomized into ‘agree’ (‘strongly agree’ and ‘agree’) and ‘disagree’ (‘disagree’, and ‘strongly disagree’).

### 3.2 Knowledge of cancer risk factors and recognition of cancer myths

**Figure 1:**
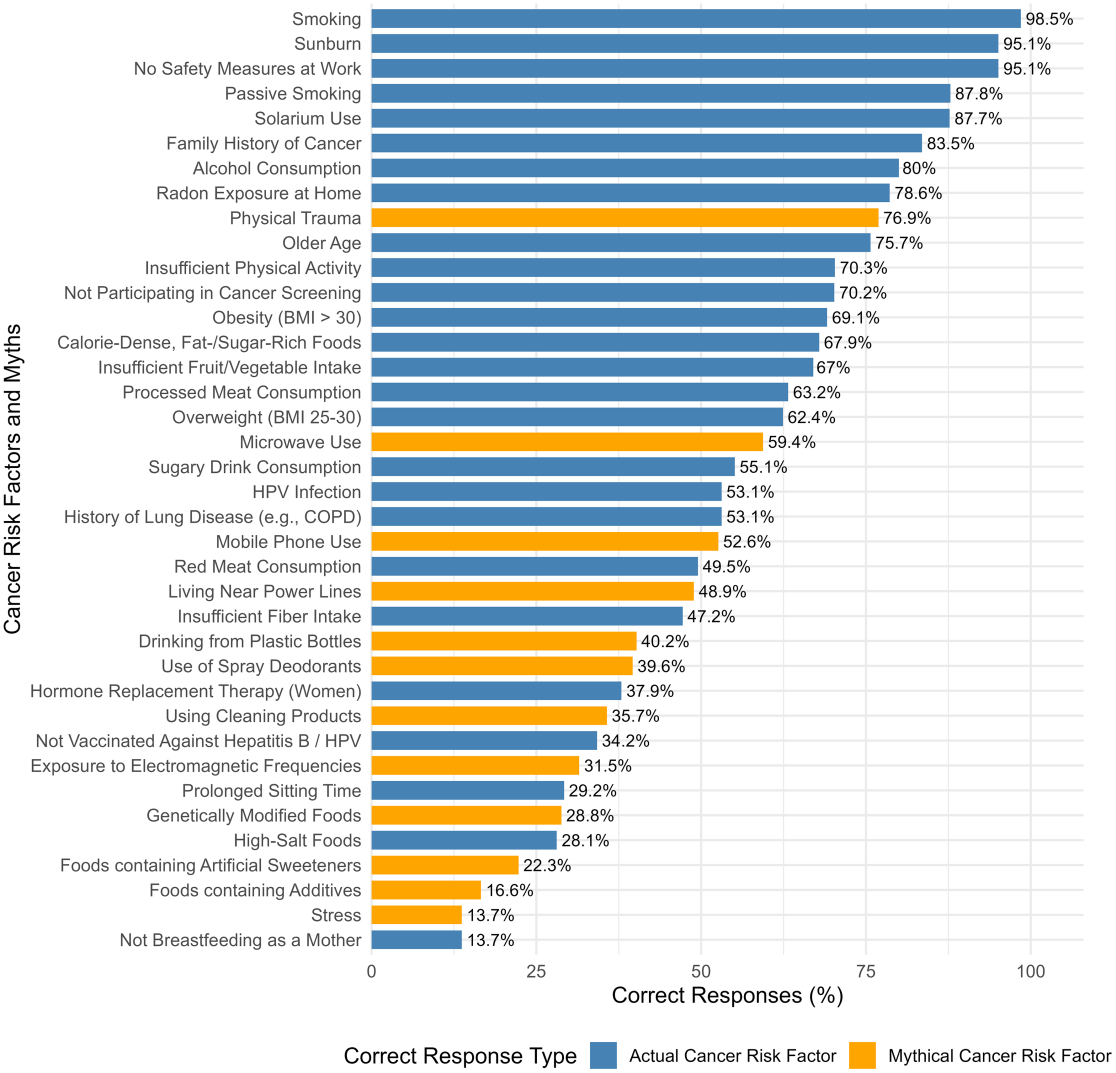
Distribution of correct responses across assessed cancer risk factors and myths.

The most commonly recognized cancer risk factors were smoking (98.5%), getting sunburned (95.1%), and not following safety regulations regarding carcinogenic substances in the workplace (95.1%). The least recognized cancer risk factors were not breastfeeding as a mother (13.7%), eating high-salt foods (28.1%), and prolonged sitting (29.2%). Of the cancer myths, physical trauma, such as a blow or bruise (76.9%), as well as microwave use (59.4%) and mobile phone use (52.6%), were most frequently identified as myths. In contrast, having stress (13.7%), eating foods with additives (16.6%) and eating foods with artificial sweeteners (22.3%) were least likely to be recognized as cancer myths. (See Figure 1).

The highest uncertainty (“do not know/unsure” responses) of the participants was observed for the following risk factors: hormone replacement therapy (in women) (46.6%), infection with HPV (39.6%), and a history of lung disease, such as chronic obstructive pulmonary disease (34.7%). (See Supplementary Figure S2).

### 3.3 Factors associated with the knowledge of actual cancer risk factors

To examine the associations between knowledge about actual cancer risk factors and several predictors, including sociodemographic factors, cancer information overload, previous information retrieval behavior, and personal cancer experience, a multiple linear regression analysis was conducted (see Table 2). Robust standard errors were used to account for heteroscedasticity. The model was statistically significant, *F*(7, 1,052) = 18.67, *p* < 0.001, explaining approximately 11.1% of the variance in knowledge scores (adjusted *R^2^* = 0.105).

**Table 2:** Multiple linear regression predicting cancer risk factors knowledge scores.

Cancer risk factors knowledge	*B*	*SE*	*β*	95% Cl		*p*
		(robust)		*LL*	*UL*	
(Intercept)	17.606	0.498	NA	16.63	18.583	< 0.001
Cancer information overload	−2.186	0.277	−0.235	−2.729	−1.644	< 0.001
Age	−0.012	0.010	−0.037	−0.032	0.008	0.234
Gender (Male)	−0.051	0.271	−0.006	−0.583	0.480	0.850
Education (middle)*	−0.713	0.310	−0.069	−1.322	−0.104	0.022
Education (low)*	−1.528	0.774	−0.053	−3.047	−0.010	0.048
Previous information retrieval	1.735	0.280	0.186	1.186	2.285	< 0.001
Personal cancer experience	0.256	0.358	0.022	−0.447	0.960	0.474

Statistically significant predictors (*p* < 0.05) are shown in bold. *Education (high) is the reference category. B = unstandardized regression coefficient; SE = standard error; *β* = standardized regression coefficient; CI = confidence interval; LL, lower limit; UL, upper limit. *N* = 1,060; with the total number differing from 1,232 due to missing data in predictor variables. Model fit: R^2^ = 0.111, Adjusted R^2^ = 0.105. Knowledge scores were calculated by summing the number of correctly identified cancer risk factors.

Cancer information overload was significantly negatively associated with knowledge scores (*β* = −0.235, *p* < 0.001). Compared to participants with high education, those in the middle group had moderately lower knowledge scores (*β* = −0.069, *p* = 0.022), and those in the low education group scored even lower (*β* = −0.053, *p* = 0.048). Given the small number of participants in the low education group included in the regression analysis (n = 28) due to missing data on other variables, and the borderline statistical significance of the observed association, this finding should be interpreted with caution. Prior information retrieval about cancer prevention was positively associated with knowledge scores (*β* = 0.186, *p* < 0.001). Age, gender, and cancer experience were no significant predictors.

### 3.4 Factors influencing the correct recognition of cancer myths

A negative binomial regression was used to assess predictors of the cancer myths knowledge score (see Table 3), accounting for overdispersion. It significantly outperformed the Poisson model (*χ^2^*(1) = 321.75, *p* < 0.001). The negative binomial regression model was statistically significant, *χ^2^*(93) = 486.25, *p* < 0.001, explaining approximately 6.4% of the variance in the cancer myths knowledge scores (Nagelkerke pseudo-*R^2^* = 0.064).

**Table 3:** Negative binomial regression predicting cancer myths knowledge scores.

Cancer myths knowledge	*B*	*SE*	*z*	*p*	Incidence Rate Ratio	95% Cl	
		(robust)				*LL*	*UL*
Intercept	1.849	0.079	23.29	< 0.001	6.351	5.422	7.412
Cancer information overload	−0.066	0.043	−1.55	0.122	0.936	0.861	1.018
Age	−0.008	0.002	−5.34	< 0.001	0.992	0.989	0.995
Gender (Male)	0.166	0.043	3.89	< 0.001	1.180	1.086	1.283
Education (middle)*	−0.048	0.048	−1.02	0.310	0.953	0.868	1.046
Education (low)*	−0.138	0.135	−1.02	0.309	0.871	0.669	1.137
Previous information retrieval	−0.042	0.044	−0.95	0.344	0.959	0.880	1.046
Personal cancer experience	0.041	0.055	0.75	0.454	1.042	0.936	1.160

Statistically significant predictors (*p* < 0.05) are shown in bold. *Education (high) is the reference category. B = log-coefficient (unstandardized); CI, confidence interval; LL, lower limit; UL, upper limit; SE, standard error. *N* = 1,060; with the total number differing from 1,232 due to missing data in predictor variables. Nagelkerke’s R^2^ (pseudo-R^2^) = 0.064. Knowledge scores were calculated by summing the number of correctly identified cancer myths.

Age was inversely associated with correct myth recognition, with each additional year linked to a 0.8% decrease in the expected score (incidence rate ratio, *IRR* = 0.992, 95% CI [0.989, 0.995], *p* < 0.001). Male participants scored significantly higher than females [*IRR* = 1.180, 95% CI (1.086, 1.283), *p* < 0.001]. Other variables including education, cancer information overload, information retrieval behavior, and personal cancer experience showed no significant association (all *p* > 0.05).

### 3.5 Relationship between knowledge of actual cancer risk factors and myths

Participants identified actual cancer risk factors (*M* = 63.6%, *SD* = 18%) significantly more accurately than cancer myths (*M* = 39.0%, *SD* = 26%), as shown by a Wilcoxon signed-rank test, V = 639,318, *p* < 0.001, with a large effect size (*ES* ≈ 0.75). Due to the highly skewed distribution of the cancer myths knowledge score, which violated normality assumptions, non-parametric tests were applied. A Spearman correlation was conducted between the knowledge score of actual cancer risk factors and the cancer myths knowledge score. The analysis revealed a significant but weak negative correlation (*ρ* = −0.11, *p* < 0.001), indicating that individuals with higher knowledge of actual cancer risk factors tended to identify myths less accurately, and vice versa. An exploratory analysis examined Spearman correlations between agreement scores on actual cancer risk factors and agreement scores on cancer myths. Here, agreement indicates participants’ endorsement of the respective items, regardless of whether the item is factually correct. This subsequent analysis revealed a significant moderate positive correlation (*ρ* = 0.34, *p* < 0.001), indicating that individuals who tended to agree more with actual cancer risk factors also tended to endorse myths more frequently.

### 3.6 Information-seeking behavior and preferences related to cancer prevention

Participants were asked whether they had ever searched for information on cancer prevention. A total of 40.5% reported having previously sought such information. For a detailed overview of sources and influencing factors, see Table 4. Participants who had previously searched for information on cancer prevention were significantly older than those who had not (Wilcoxon rank-sum test: *W* = 133,095, *p* < 0.001). Among those with prior search experience, the internet was the most frequently used source followed by healthcare professionals. Additionally reported sources included books, television, health insurance providers, and workplace-related training. Key factors influencing participants’ use of the information found included expert production or review and affiliation with a government agency or university.

**Table 4:** Actual and hypothetical information-seeking behavior related to cancer prevention.

Sources/Influencing factors	Past information retrieval (%)	Possible future information retrieval (%)
	(*N* = 498)	(*N* = 651)
Information sources		
Internet	80.3	86.6
via search engines	97.0	96.8
via online forums	17.8	23.9
via social media platforms	10.5	8.3
Healthcare professionals	56.8	68.0
Brochures	40.2	25.3
Family and friends	33.7	33.3
Cancer information service	9.4	22.4
Other	5.6	1.2
Factors influencing (potential) use		
Expert production or review	65.5	82.6
Government agency or university affiliation	53.4	71.7
Trust in the information source	39.8	73.4
Ease of access	40.4	63.7
Author’s qualifications	36.1	60.7
Recommendation by others	19.5	37.9
Appealing design/graphics	9.2	23.5
First result encountered	6.4	15.2
Other	0.4	0.9

Participants were first asked whether they had ever sought information on cancer prevention. Those who answered “yes” were asked which sources they had used and which features influenced their choice. Those who answered “no” were asked which sources they would consider using and which features would influence their choice. Multiple responses were permitted for all questions except the initial yes/no question.

In contrast, 52.9% of participants had never actively searched for such information. Still, their stated preferences largely mirrored those of previous searchers, with the internet and healthcare professionals being the most preferred sources. The only larger discrepancy was found regarding the Cancer Information Service of the German Cancer Research Center, which is a national source providing reliable, evidence-based information on cancer to patients, families, and the public via phone, email, and online. While 22.4% of participants stated they would hypothetically use the Cancer Information Service, only 9.4% reported using it. Differences were also observed regarding factors potentially influencing information use, with participants considering possible future use generally selecting more influencing factors than those who had actually searched in the past reported. Facilitators for future information use included expert production or review and trust in the information source. Open-text responses revealed additional criteria, including easily understandable and well-structured content, absence of advertisements, and concise and accessible presentation.

## 4 Discussion

Our results reveal varying levels of awareness regarding evidence-based and mythical cancer risk factors within our sample. Consistent with prior research (8, 9, 11), nearly all participants recognized smoking as a cancer risk factor. Passive smoking was also widely acknowledged within our study, aligning with some studies reporting high recognition (11, 13, 31) but contrasting with others indicating lower awareness (12).

Compared to other studies (7, 9, 12), our findings demonstrated greater awareness of alcohol consumption and physical inactivity as cancer risk factors. Cultural context may partly explain these difference: for instance, in Canada (9), public alcohol consumption is prohibited and consumption is strictly regulated, which can create the impression that the risk factor alcohol is under control, potentially reducing personal risk awareness. Similarly, in Malaysia (7), where the majority of the population is Muslim and therefore alcohol consumption is largely prohibited, awareness of alcohol-related cancer risks might be similarly low due to limited alcohol exposure.

Awareness of overweight and obesity risks in our sample was comparable to other European populations (31, 40) but higher than in African (11) and Asian samples (7, 10, 13). These differences may partly reflect cultural and socioeconomic contexts. In Saudi Arabia (10) and Malaysia (7), countries with a high Human Development Index (HDI) (41) and overweight/obesity rates (42, 43) similar to or exceeding those in Europe (44), changes in dietary patterns may outpace public recognition of associated risks, particularly for factors related to high fat and sugar intake. By contrast, in Ethiopia (11) and Pakistan (13), both lower-HDI countries, limited health education and access to reliable information, especially in rural areas, likely contribute to reduced awareness. For dietary factors, awareness of insufficient fruit and vegetable intake was higher in our sample than in most others reviewed (7, 9, 10, 40), where fewer than half of participants recognized this risk factor, with the exception of the Ethiopian sample (11) whose awareness levels were similar to ours. In contrast, awareness of red and processed meat consumption was not elevated in our sample, aligning with ranges in previous studies (7, 9, 11, 31). Processed meat consumption was recognized as a cancer risk factor substantially more often than red meat (63.2% vs. 49.5%), highlighting differences in public awareness of specific dietary risks. Overall, these findings suggest that dietary factors are broad and complex, and that socioeconomic and cultural influences may differentially shape awareness of specific dietary risks. Although some risk factors were more frequently recognized in our sample, overall awareness remained moderate, underscoring the need for continued public education.

Awareness was considerably lower for several factors included in the ECAC4, such as lack of Hepatitis B or HPV vaccination, not breastfeeding, prolonged sitting, and high-salt food consumption. The latter three were identified by fewer than one-third of respondents. Additionally, the greatest uncertainty, as indicated by frequent “don’t know/not sure” responses, was observed regarding hormone replacement therapy in women and HPV infection, emphasizing a critical need for enhanced medical education.

This uncertainty also extended to mythical cancer risk factors. Compared to prior research (7), our participants showed particularly high uncertainty regarding mythical cancer risks - especially nutrition-related misconceptions, such as genetically modified foods and foods with additives. Of the twelve mythical factors assessed, only mobile phone use, microwave use, and physical trauma were correctly identified as myths by more than half of participants, with recognition largely consistent with prior research (9).

When examining factors associated with knowledge of evidence-based cancer risk factors, our findings suggest that lower levels of cancer information overload, higher educational attainment, and prior information retrieval are associated with higher knowledge of evidence-based cancer risk factors. Notably, while in line with previous research (7, 10, 11) knowledge was generally lower among participants with lower educational attainment, the association for the low education group was only marginally significant and based on a small sample size, warranting cautious interpretation. Age, gender, and personal cancer experience showed no significant influence on the knowledge score. While earlier studies reported a negative relation between older age and risk factor knowledge (11, 13), evidence regarding the effect of personal cancer experience is inconsistent, with some finding no association with cancer prevention knowledge (13, 45), whereas others reported that it increases the likelihood of correctly identifying risk factors (7).

Conversely, the factors associated with knowledge of evidence-based cancer risk factors, education, cancer information overload, and previous information retrieval were not significantly related to myth recognition, suggesting that higher education levels or prior engagement with cancer prevention information were not related to believing in or feeling unsure about cancer myths. Instead, only age and gender showed significant associations: older participants were less likely to correctly identify myths, while males had a higher likelihood of doing so. Other studies provided mixed evidence on factors influencing correct myth recognition. Studies from Canada and England (9, 31) also found a negative association between correct myth identification and increasing age, and, similarly, research in the English and Malaysian populations (7, 31) reported that females were more likely to endorse mythical factors. In contrast, a study in Pakistan observed no influence of age or gender on myth recognition (13).

We found that participants were significantly better at detecting correct cancer risk factors than cancer myths, a finding that is in line with previous research (9, 13, 31). Furthermore, individuals with higher knowledge of real cancer risk factors tended to identify myths less accurately, and vice versa, a finding that has been reported before (7, 13). In further exploration, we found that individuals who tended to agree more with actual cancer risk factors also tended to endorse myths more frequently. This appears to reflect a general tendency among some participants to assume that a wide range of factors can cause cancer.

In our sample, 40.5% of participants reported having previously searched for information on cancer prevention, a slightly lower proportion than in earlier studies (7, 46), which examined the broader topic of cancer-related information-seeking. Consistent with previous research conducted in a Japanese population (27), older participants in our study were more likely to seek cancer prevention information. In line with these findings, the internet and healthcare professionals emerged as the most frequently used sources. Expert production or review, as well as affiliation with a government agency or university, were the most influential factors guiding information use, likely due to their perceived credibility, which has been revealed to be crucial in fostering cancer prevention behaviors (47). Likewise, it has been found that reliable sources, such as health professionals and public institution websites, were associated with a range of favorable health behaviors, including dietary improvement (27). Preferences among non-seekers largely mirrored those of seekers, suggesting similar information needs across groups. Since the internet was the preferred source for all participants, and existing research showed potential to decrease cancer information overload by engaging in online health information-seeking, particularly when finding useful information on cancer (17, 25, 48), providing easily accessible, trustworthy online resources appears essential to support effective cancer prevention. This is particularly critical given that 44.6% of our participants reported experiencing cancer information overload. Notably, while the Cancer Information Service - an expert-curated online resource - was hypothetically valued by many, actual reported use was rare. This suggests that most people were simply unaware of it, highlighting the need to increase its visibility in the future.

This study has several strengths and limitations. First, participants were randomly selected from the residents’ registration office, and data quality was ensured by seriousness checks. The CAM was expanded to include ECAC4-recommended risk factors, thereby enabling a more comprehensive assessment. Moreover, offering both online and paper-and-pencil formats improved accessibility. However, the underrepresentation of individuals with lower education may limit generalizability. In addition, self-reported survey data may be subject to social desirability bias and recall bias, although the anonymous survey design may have reduced socially desirable responding. Additionally, cancer information overload was assessed using a single item, which may reduce measurement precision, although this approach has been used in previous studies (21, 37). Furthermore, the assessment of cancer prevention-related information behavior focused exclusively on active information seeking and did not capture passive exposure to cancer prevention information through sources such as public health campaigns, healthcare professionals, or mass media. Consequently, participants classified as not having sought information may nevertheless have been exposed to relevant cancer prevention information. Finally, the cross-sectional design limits causal interpretation, and the requirement for sufficient German language proficiency excluded individuals without these skills.

### 4.1 Conclusion

Our findings highlight substantial gaps in public knowledge of many modifiable cancer risk factors in Germany, coupled with widespread endorsement of or uncertainty about common cancer myths. Improving access to expert-reviewed information that is perceived as clear and trustworthy - especially via commonly used sources like the internet and healthcare professionals - could improve public understanding, reduce cancer information overload, and promote preventive behaviors. Education efforts should prioritize medical and dietary factors with particularly low awareness, such as high-salt foods, HPV infection and vaccination, as well as protective factors like breastfeeding, and directly address prevalent misconceptions. Given the observed association between educational attainment and cancer risk factor knowledge, information on these topics should be introduced early in school health curricula so that all students, regardless of their later educational path, have the opportunity to acquire essential prevention knowledge. Complementary public campaigns and counseling opportunities in healthcare settings may further support the dissemination of evidence-based cancer prevention information and reinforce these messages throughout life, thereby supporting cancer risk reduction at the population level.

## Data Availability

The raw data supporting the conclusions of this article will be made available by the authors on reasonable request.

## References

[1] Bray F Laversanne M Sung H Ferlay J Siegel RL Soerjomataram I . Global Cancer statistics 2022: Globocan estimates of incidence and mortality worldwide for 36 cancers in 185 countries. CA Cancer J Clin. (2024) 74:229–63. doi: 10.3322/caac.21834, 38572751

[2] Zentrum für Krebsregisterdaten. Krebs Gesamt: Robert Koch-Institut (2025). Available online at: https://www.krebsdaten.de/Krebs/DE/Content/Krebsarten/Krebs_gesamt/krebs_gesamt_node.html (Accessed November 13, 2025).

[3] Statistisches Bundesamt (Destatis). Todesursachen 2024: Immer Mehr Menschen Versterben an Demenz (Pressemitteilung Nr. 377): Statistisches Bundesamt (Destatis). (2025). Available online at: https://www.destatis.de/DE/Presse/Pressemitteilungen/2025/10/PD25_377_23211.html?templateQueryString=krebs (Accessed November 13, 2025).

[4] Gredner T Behrens G Stock C Brenner H Mons U. Cancers due to infection and selected environmental factors. Dtsch Arztebl Int. (2018) 115:586–93. doi: 10.3238/arztebl.2018.0586, 30236218 PMC6206252

[5] Fink H Langselius O Vignat J Rumgay H Rehm J Martinez RX . Global and regional Cancer burden attributable to modifiable risk factors to inform prevention. Nat Med. (2026). doi: 10.1038/s41591-026-04219-7 41634393

[6] Schüz J Espina C Villain P Herrero R Leon ME Minozzi S . European code against Cancer 4th edition: 12 ways to reduce your Cancer risk. Cancer Epidemiol. (2015) 39:S1–S10. doi: 10.1016/j.canep.2015.05.009, 26164654

[7] Bujang N-N-A Kong Y-C Danaee M Munisamy M Kaur R Rajah HDA . Beliefs on causes of Cancer in the general population, and the association with risk perception and lifestyle in a multiethnic setting. JCO Glob Oncol. (2024) 10:e2400129. doi: 10.1200/GO.24.00129, 39509673 PMC11583347

[8] Paunescu A-C Delpierre C Jacob G Delrieu L Pannard M Préau M . Compliance with public health recommendations of Cancer-free female research volunteers: the French Seintinelles study. Cancer Causes Control. (2024) 35:293–309. doi: 10.1007/s10552-023-01788-7, 37733136

[9] Rydz E Telfer J Quinn EK Fazel SS Holmes E Pennycook G . Canadians' knowledge of Cancer risk factors and belief in Cancer myths. BMC Public Health. (2024) 24:329. doi: 10.1186/s12889-024-17832-3, 38291409 PMC10829248

[10] Sabi EM Mujamammi AHA Abdulghani M Almesfer YM Alsuwaida AA Balobaid AS . Awareness level of cancer risk factors and warning signs and cancer campaign attendance behavior among Saudi adults in a tertiary Hospital in Riyadh. Asian Pac J Cancer Prev. (2021) 22:2421–8. doi: 10.31557/APJCP.2021.22.8.2421, 34452554 PMC8629485

[11] Tekeste Z Berhe N Arage M Degarege A Melaku YA. Cancer signs and risk factors awareness in Addis Ababa, Ethiopia: a population-based survey. Infect Agent Cancer. (2023) 18:1. doi: 10.1186/s13027-022-00477-5, 36600261 PMC9811709

[12] Union for International Cancer Control. International Public Opinion Survey on Cancer. Geneva: Union for International Cancer Control (2020). Available online at: https://www.uicc.org/sites/default/files/atoms/files/WCD20_IntPublicOpinionPoll_Report_FA_Screen_0.pdf (Accessed July 08, 2025).

[13] Munir R Noureen N Bashir M Shoaib N Ashraf A Lisec J . Cancer awareness measure (cam) and Cancer awareness measure mythical causes scale (cam-my) scores in Pakistani population. Sci Rep. (2022) 12:8887. doi: 10.1038/s41598-022-13012-8, 35614124 PMC9132919

[14] Ahmed SBM Amer S Hussein A Kampani DD Al Hasham N Assker MM . Assessing the knowledge of environmental risk factors for Cancer among the Uae population: a pilot study. Int J Environ Res Public Health. (2020) 17:2984. doi: 10.3390/ijerph17092984, 32344867 PMC7246594

[15] Lizama N Jongenelis M Slevin T. Awareness of Cancer risk factors and protective factors among Australian adults. Health Promot J Austr. (2020) 31:77–83. doi: 10.1002/hpja.248, 30932242

[16] Jensen JD Carcioppolo N King AJ Scherr CL Jones CL Niederdieppe J. The Cancer information overload (Cio) scale: establishing predictive and discriminant validity. Patient Educ Couns. (2014) 94:90–6. doi: 10.1016/j.pec.2013.09.016 24268921

[17] Serçekuş P Gencer H Özkan S. Finding useful Cancer information may reduce Cancer information overload for internet users. Health Inf Libr J. (2020) 37:319–28. doi: 10.1111/hir.12325, 32770732

[18] Shi W Rothman AJ Yzer MC Nagler RH. Effects of exposure to conflicting information about mammography on Cancer information overload, perceived scientists' credibility, and perceived journalists' credibility. Health Commun. (2023) 38:2481–90. doi: 10.1080/10410236.2022.2077163, 35607276 PMC9681936

[19] Zheng Y Wang JZ Zhu Y Zhao X. Effect of E-health use on Cancer screening mediated through Cancer worry and fatalism: a cross-sectional study of older adults. Cancer Control. (2025) 32:10732748251355831. doi: 10.1177/10732748251355831, 40619345 PMC12230283

[20] Zaidi M Sarkar S Arakelyan S Poghosyan H. Relationship between fatalistic Cancer beliefs and risky health behaviors. West J Nurs Res. (2024) 46:757–65. doi: 10.1177/01939459241273388, 39161288

[21] Jensen JD Shannon J Iachan R Deng Y Kim SJ Demark-Wahnefried W . Examining rural-urban differences in fatalism and information overload: data from 12 Nci-designated Cancer centers. Cancer Epidemiol Biomarkers Prev. (2022) 31:393–403. doi: 10.1158/1055-9965.EPI-21-0355, 35091459 PMC9035270

[22] Fleary SA Paasche-Orlow MK Joseph P Freund KM. The relationship between health literacy, Cancer prevention beliefs, and Cancer prevention behaviors. J Cancer Educ. (2019) 34:958–65. doi: 10.1007/s13187-018-1400-2, 30022378 PMC6339599

[23] Jensen JD Pokharel M Carcioppolo N Upshaw S John KK Katz RA. Cancer information overload: discriminant validity and relationship to sun safe behaviors. Patient Educ Couns. (2020) 103:309–14. doi: 10.1016/j.pec.2019.08.039, 31522897 PMC7012722

[24] Bhandari D Ozaki A Kobashi Y Higuchi A Shakya P Tanimoto T. Cancer information seeking and scanning behavior among Nepalese migrants in Japan and its association with preventive behavior. PLoS One. (2020) 15:e0235275. doi: 10.1371/journal.pone.0235275, 32598343 PMC7347024

[25] Chae J Lee C-j Jensen JD. Correlates of Cancer information overload: focusing on individual ability and motivation. Health Commun. (2016) 31:626–34. doi: 10.1080/10410236.2014.986026, 26512760

[26] Jensen JD Liu M Carcioppolo N John KK Krakow M Sun Y. Health information seeking and scanning among us adults aged 50-75 years: testing a key postulate of the information overload model. Health Informatics J. (2017) 23:96–108. doi: 10.1177/1460458215627290, 26905079

[27] Yamagiwa Y Tanaka S Abe SK Shimazu T Inoue M. A cross-sectional survey on awareness of Cancer risk factors, information sources and health behaviors for Cancer prevention in Japan. Sci Rep. (2022) 12:14606. doi: 10.1038/s41598-022-18853-x, 36028524 PMC9418251

[28] Redmond N Baer HJ Clark CR Lipsitz S Hicks LS. Sources of health information related to preventive health behaviors in a National Study. Am J Prev Med. (2010) 38:620–7.e2. doi: 10.1016/j.amepre.2010.03.001, 20494238 PMC2885154

[29] Swoboda CM Walker DM Huerta T. Odds of meeting Cancer prevention behavior recommendations by health information seeking behavior: a cross-sectional hints analysis. J Cancer Educ. (2021) 36:56–64. doi: 10.1007/s13187-019-01597-0, 31396847

[30] Statistics UIf. International Standard Classification of Education: Isced 2011. Montreal: UNESCO Institute for Statistics (2012).

[31] Shahab L McGowan JA Waller J Smith SG. Prevalence of beliefs about actual and mythical causes of Cancer and their association with socio-demographic and health-related characteristics: findings from a cross-sectional survey in England. Eur J Cancer. (2018) 103:308–16. doi: 10.1016/j.ejca.2018.03.029, 29705530 PMC6202672

[32] World Health Organization. Healthy Diet Geneva. (2026). Available online at: https://www.who.int/news-room/fact-sheets/detail/healthy-diet (Accessed March 10, 2026).

[33] World Health Organization. Who Guidelines on Physical Activity and Sedentary Behaviour. Geneva: World Health Organization (2020). Available online at: https://iris.who.int/server/api/core/bitstreams/faa83413-d89e-4be9-bb01-b24671aef7ca/content (Accessed March 10, 2026).

[34] Connor K Hudson B Power E. Awareness of the signs, symptoms, and risk factors of Cancer and the barriers to seeking help in the Uk: comparison of survey data collected online and face-to-face. JMIR Cancer. (2020) 6:e14539. doi: 10.2196/14539, 31951219 PMC6996748

[35] Stubbings S Robb K Waller J Ramirez A Austoker J Macleod U . Development of a measurement tool to assess public awareness of Cancer. Br J Cancer. (2009) 101 Suppl 2:S13–7. doi: 10.1038/sj.bjc.6605385, 19956157 PMC2790699

[36] Smith SG Beard E McGowan JA Fox E Cook C Pal R . Development of a tool to assess beliefs about mythical causes of Cancer: the Cancer awareness measure mythical causes scale. BMJ Open. (2018) 8:e022825. doi: 10.1136/bmjopen-2018-022825, 30552257 PMC6303629

[37] Tatum KL Morris BB Glasgow TE Lee SMJ Barsell DJ Fugate-Laus K . Rural-specific identity and associations with lifestyle behaviors and well-being among rural Cancer survivors. J Rural Health. (2024) 40:623–33. doi: 10.1111/jrh.12835, 38556709 PMC13370668

[38] Finney Rutten LJ Blake KD Skolnick VG Davis T Moser RP Hesse BW. Data resource profile: the National Cancer Institute's health information National Trends Survey (hints). Int J Epidemiol. (2020) 49:17–17j. doi: 10.1093/ije/dyz083, 31038687 PMC7124481

[39] R. Core Team. *R*. Vienna, Austria: R Foundation for Statistical Computing (2024).

[40] Lagerlund M Hvidberg L Hajdarevic S Fischer Pedersen A Runesdotter S Vedsted P . Awareness of risk factors for Cancer: a comparative study of Sweden and Denmark. BMC Public Health. (2015) 15:1156. doi: 10.1186/s12889-015-2512-9, 26596679 PMC4655457

[41] United Nations Development Programme. Human Development Report 2025. New York: United Nations (2025). Available online at: https://hdr.undp.org/content/human-development-report-2025 (Accessed September 05, 2025).

[42] Lee YY Wan Muda WAM. Dietary intakes and obesity of Malaysian adults. Nutr Res Pract. (2019) 13:159–68. doi: 10.4162/nrp.2019.13.2.159, 30984360 PMC6449549

[43] Alqarni MSS. A review of prevalence of obesity in Saudi Arabia. J Obes Eat Disord. (2016) 2:25. doi: 10.21767/2471-8203.100025

[44] Marques A Peralta M Naia A Loureiro N Matos MG. Prevalence of adult overweight and obesity in 20 European countries, 2014. Eur J Pub Health. (2018) 28:295–300. doi: 10.1093/eurpub/ckx143, 29036436

[45] Karasiewicz M Chawłowska E Lipiak A Wiȩckowska B. How to improve Cancer prevention knowledge? A way to identify gaps and tackle the limited availability of health education Services in Primary Health Care Using the European code against Cancer. Front Public Health. (2022) 10:878703. doi: 10.3389/fpubh.2022.878703, 35586014 PMC9109786

[46] Kobayashi LC Smith SG. Cancer fatalism, literacy, and Cancer information seeking in the American public. Health Educ Behav. (2016) 43:461–70. doi: 10.1177/1090198115604616, 26377524 PMC5123630

[47] Issaka B Allor P Aidoo EAK Wood SF Amissah AA Muturi N. Enhancing Cancer prevention behaviors through social media: the role of source credibility and message characteristics in Ghana. BMC Public Health. (2025) 25:2471. doi: 10.1186/s12889-025-23693-1, 40670961 PMC12265361

[48] Wu Y Zhang L Zhao X. Linking online health information seeking to Cancer information overload among Chinese Cancer patients' family members. Digit Health. (2025) 11:20552076251336308. doi: 10.1177/20552076251336308, 40297380 PMC12035502

