# Supplementary material for: Cancer prevention in Germany: awareness, beliefs, and information-seeking behaviors in a population-based survey
Source: Front Public Health. 2026 Jun 30;14:1868556. doi: 10.3389/fpubh.2026.1868556 (PMC13365034; doi:10.3389/fpubh.2026.1868556)
Supplement: Supplementary file 1 [file Data_Sheet_1.docx]

Supplementary Material

# Supplementary Methods

Supplement 1. Sampling strategy and Participant Recruitment

When contacting participants, the CLARO study aimed to achieve a high response rate and an adequate representation of individuals with a migration history, lower income and lower education achievement. As an additional measure to enhance the representation of individuals from socially and economically disadvantaged groups, we implemented oversampling within city boroughs. Previous studies showed that individuals with the lowest levels of educational attainment tend to participate at considerably lower rates (1, 2). Therefore, we oversampled by 23% (*N* = 1,380) to target underrepresented groups. The remaining 77% of the sampling (*N* = 4,620) reflected the general population distribution across the city boroughs.

In general, Stuttgart is divided into a total of 152 city boroughs, which are grouped into 23 city districts. Oversampling was targeted at city boroughs characterized by a high proportion of residents with a migration background, a high proportion of unemployment benefits II recipients, and a lower average income levels. These three indicators were obtained from the official website of Stuttgart’s Statistical Office (3). Specifically, the office provided borough-level data on the averaged individual income (from 2019), the proportion of unemployment benefit II recipients (from 2022), and the proportion of residents with a migration background (from 2022). Each of indicator was z-transformed and the resulting values were averaged to create a composite index of socioeconomic disadvantage.

Due to the small number of inhabitants, there was no available data for 10 city boroughs. Three boroughs - namely the university borough, Hohenheim, and Pfaffenwald - were excluded from the oversampling approach, despite their low income and high migration rates, due to their disproportionately high student populations, which did not align with the intended oversampling focus. In total, we oversampled 1,380 individuals across 57 city boroughs, with the number of additional individuals per borough ranging from 2 to 61.

Although the preregistration initially specified contacting 5,800 individuals, an additional 200 letters were sent due to a higher-than-expected number of undeliverable returned mail.

References

1. Vo CQ, Samuelsen P-J, Sommerseth HL, Wisløff T, Wilsgaard T, Eggen AE. Comparing the Sociodemographic Characteristics of Participants and Non-Participants in the Population-Based Tromsø Study. *BMC Public Health* (2023) 23(1):994. doi: 10.1186/s12889-023-15928-w.

2. Christensen AI, Lau CJ, Kristensen PL, Johnsen SB, Wingstrand A, Friis K, et al. The Danish National Health Survey: Study Design, Response Rate and Respondent Characteristics in 2010, 2013 and 2017. *Scand J Public Health* (2022) 50(2):180-8. doi: 10.1177/1403494820966534.

3. Statistikamt S. *Sozialatlas Stuttgart – Stadtteile*. (n.d.).

# Supplementary Figures

##
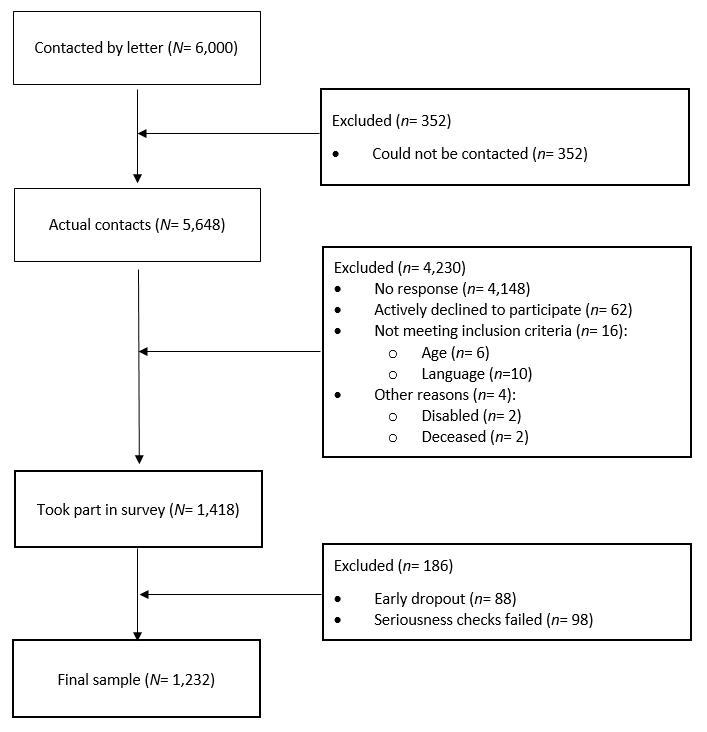


## Supplementary Figure 1.

*Participation flow-chart.*


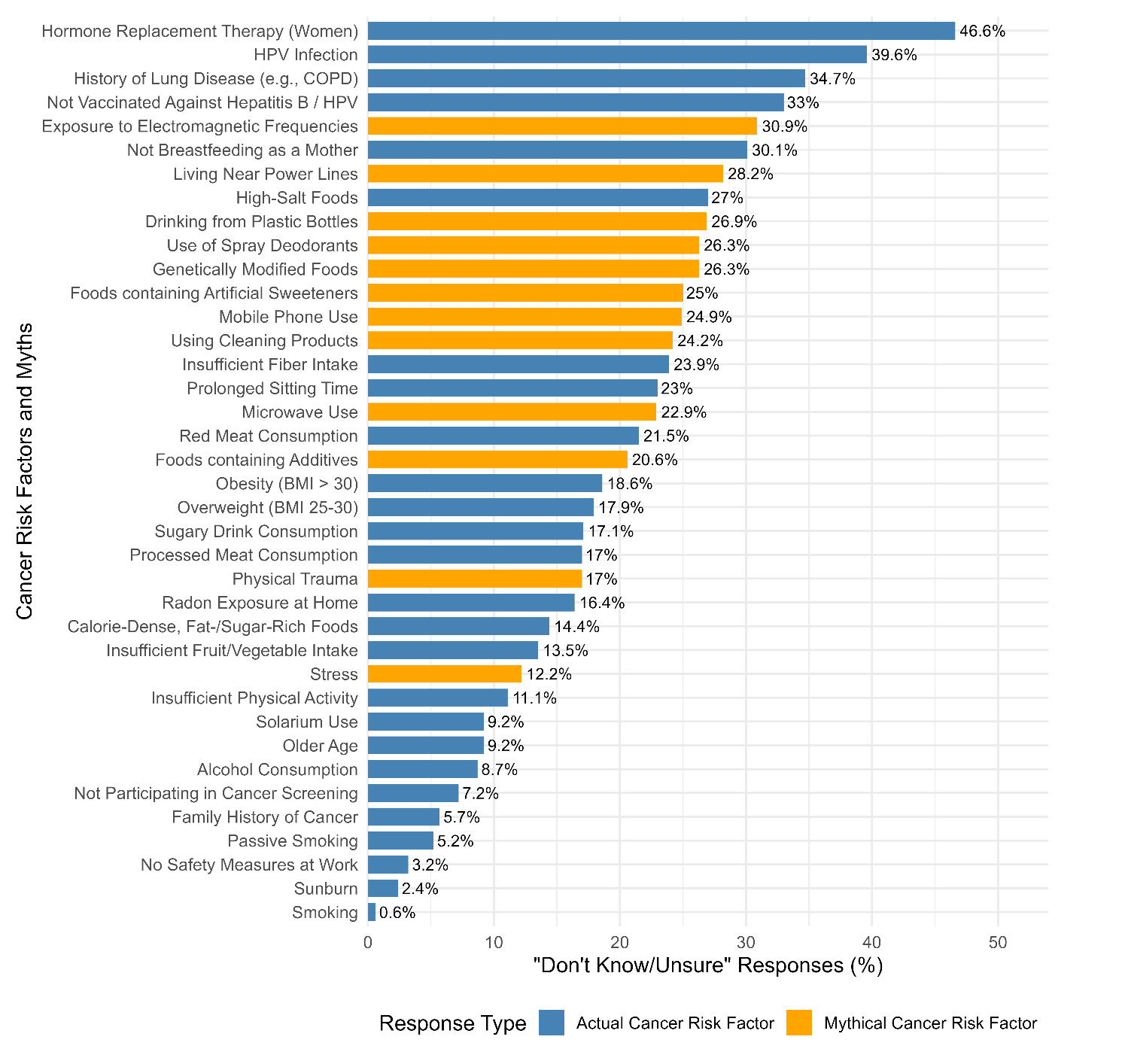


## Supplementary Figure 2.

*Distribution of “Don’t know/unsure” responses across assessed cancer risk factors and myths.*
